# Effect of Anisotropic Electrical Conductivity Induced by Fiber Orientation on Ablation Characteristics of Pulsed Field Ablation in Atrial Fibrillation Treatment: A Computational Study

**DOI:** 10.3390/jcdd9100319

**Published:** 2022-09-22

**Authors:** Lianru Zang, Kaihao Gu, Xingkai Ji, Hao Zhang, Shengjie Yan, Xiaomei Wu

**Affiliations:** 1 Center for Biomedical Engineering, School of Information Science and Technology, Fudan University, Shanghai 200438, China; 2 Academy for Engineering and Technology, Fudan University, Shanghai 200433, China; 3 Key Laboratory of Medical Imaging Computing and Computer-Assisted Intervention (MICCAI) of Shanghai, Fudan University, Shanghai 200032, China; 4 Shanghai Engineering Research Center of Assistive Devices, Shanghai 200093, China; 5 Yiwu Research Institute, Fudan University, Yiwu 322000, China

**Keywords:** pulsed field ablation, atrial fibrillation, fiber orientation, anisotropic electrical conductivity, finite element analysis

## Abstract

Pulsed field ablation (PFA) is a promising new ablation modality for the treatment of atrial fibrillation (AF); however, the effect of fiber orientation on the ablation characteristics of PFA in AF treatment is still unclear, which is likely an essential factor in influencing the ablation characteristics. This study constructed an anatomy-based left atrium (LA) model incorporating fiber orientation and selected various electrical conductivity and ablation targets to investigate the effect of anisotropic electrical conductivity (AC), compared with isotropic electrical conductivity (IC), on the ablation characteristics of PFA in AF treatment. The results show that the percentage differences in the size of the surface ablation area between AC and IC are greater than 73.71%; the maximum difference in the size of the ablation isosurface between AC and IC at different locations in the atrial wall is 3.65 mm (*X*-axis), 3.65 mm (*Z*-axis), and 4.03 mm (*X*-axis), respectively; and the percentage differences in the size of the ablation volume are greater than 6.9%. Under the condition of the pulse, the amplitude is 1000 V, the total PFA duration is 1 s, and the pulse train interval is 198.4 ms; the differences in the temperature increase between AC and IC in LA are less than 2.46 °C. Hence, this study suggests that in further exploration of the computational study of PFA in AF treatment using the same or similar conditions as those used here (myocardial electrical conductivity, pulse parameters, and electric field intensity damage threshold), to obtain more accurate computational results, it is necessary to adopt AC rather than IC to investigate the size of the surface ablation area, the size of the ablation isosurface, or the size of the ablation volume generated by PFA in LA. Moreover, if only investigating the temperature increase generated by PFA in LA, adopting IC instead of AC for simplifying the model construction process is reasonable.

## 1. Introduction

Atrial fibrillation (AF) is one of the most common arrhythmias in the clinic. Aside from medication, left atrium (LA) radiofrequency ablation (RFA) is currently the gold standard for AF clinical treatment, which works by destroying the local myocardium to isolate abnormal electrical signals causing arrhythmia. However, RFA still has important limitations, exceptionally high recurrence rates, significant complication rates, and lengthy procedure times [1]. Pulsed field ablation (PFA) is a promising new non-thermal ablation modality based on cell membrane irreversible electroporation (IRE), which is applied to the myocardium and leads to disruptions in the cellular membrane integrity and cell homeostasis, eventually resulting in cell apoptosis and replacement fibrosis. The promise of PFA is the attainment of highly efficient ablation procedures with short procedural times; the creation of continuous, transmural ablations; and an enhanced safety profile with minimal to no collateral damage [2].

With the rapid development of PFA in AF treatment in recent years, several computational studies based on 3D medical imaging reconstruction combined with finite element analysis (FEA) have emerged to investigate the ablation characteristics generated by PFA in the pulmonary veins (PVs), in combination with experimental results [3] or comparing the safety and efficacy of different PFA catheters [4]. However, almost all relevant computational studies simplified the LA model to some extent to shorten the model construction process and reduce the computational complexity; one of the significant simplifications is to define the electrical conductivity of the LA model as isotropic electrical conductivity (IC). In fact, the realistic LA has a complex fiber architecture, and electrical conductivity is higher in fiber orientation than transversally. In general, for normal myocardium, the longitudinal to transverse ratio of myocardial electrical conductivity ranges from 1.66 (1 Hz–1 kHz) [5]–3.75 [6], and for myocardium with AF, the ratio ranges from 4.98 [7]–10 [8]. This anisotropic electrical conductivity (AC) induced by fiber orientation affects the electric field distribution generated by PFA in LA, and PFA inducing myocardial cell destruction mainly depends on the applied electric field intensity [9]; therefore, AC may affect the ablation characteristics of PFA in AF treatment and simplify the electrical conductivity, as IC in the LA model may not reflect the actual ablation results as consistently as possible.

Although it is reasonable to consider the electrical conductivity of the LA model as AC in the computational study of PFA in AF treatment, incorporating fiber orientation into the LA model has so far been complex and time consuming. In a review of relevant studies, AC induced by fiber orientation on the ablation characteristics of PFA in AF treatment was not investigated. Accordingly, this study investigates in the computational study of PFA in AF treatment (1) the effect of AC compared with IC on ablation characteristics; (2) the effect of AC with a different size and anisotropy ratio on ablation characteristics; the objective is to provide reasonable advice on which electrical conductivity to choose in further studies, in order to achieve the optimal choice of obtaining results that are as consistent as possible with actual ablation, or to simplify the model construction process without compromising the reasonableness of the results.

## 2. Materials and Methods

### 2.1. Model Construction

**Figure 1:**
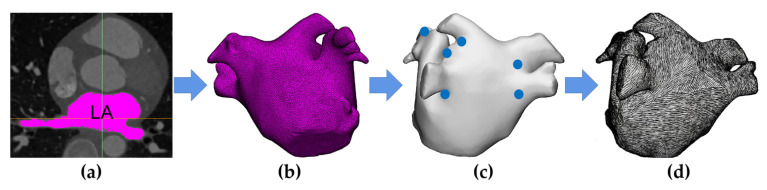
A flow chart of the processes of constructing an anatomy-based model of the left atrium (LA) incorporating fiber orientation. (**a**) Segment LA region from coronary computed tomography angiography (CTA) images; (**b**) generate the triangular and tetrahedral meshes of LA model; (**c**) manually mark seeds on the LA model to incorporate fiber orientation into the LA model; (**d**) LA model incorporating fiber orientation.

Figure 1 shows the process of constructing an anatomy-based model of LA incorporating fiber orientation. The process was divided into two parts: firstly, reconstructing the LA model by coronary computed tomography angiography (CTA) images (Figure 1a,b), and then using a robust semi-automatic algorithm to incorporate fiber orientation into the LA model (Figure 1c,d).

CTA images of a female patient with persistent AF provided by the hospital were used to reconstruct the LA model. CTA scans were performed using a SOMATOM Drive (Siemens, Berlin and Munich, Germany); the number of slices is 298, the slice resolution is 512 × 512 pixels, the slice thickness is 0.75 mm, and the pixel size is 0.326172 mm.

Semi-automatic segmentation of the LA region from CTA images was performed using CemrgApp v2.2 (Figure 1a) [10]. The LA region was defined as the pixels contained within the LA endocardial surface, including the PVs, the mitral valve (MV), as well as the LA appendage (LAA) [11]. Due to the MV showing a similar image intensity as LA and the left ventricle, a cutting plane was manually inserted in the position of the MV to mark seed points at the edge of the MV in subsequent steps. Then, region growing was used to separate the LA region from the selected slices; to reduce the impact of noise or image artefacts on the segmentation outcome, details were manually corrected. The resulting segmentations were exported as a triangular mesh.

A segmentation of the LA epicardial surface from traditional CTA images is usually not feasible due to an insufficient spatial resolution and a limited signal-to-noise ratio. To build the epicardial surface, this study assumes that the thickness of the LA wall is homogeneous; the endocardial surface of the mesh was dilated by 2.4 mm at each point along the normal direction, which is the mean measured wall thickness of the human LA [12]. To build the MV, this study set a circle with a diameter of 24 mm at the bottom of the LA model [13]. Then, a double-layer triangular mesh was transformed into a tetrahedral mesh with a mean edge length of 400 µm using Gmsh v4.10.1 [14]; this resolution falls within the range required for numerical convergence (Figure 1b) [15].

To incorporate fiber orientation into the LA model, 13 seed points corresponding to specific anatomical positions were manually marked on the LA model (Figure 1c). Then, fiber orientation was annotated by a semi-automatic rule-based algorithm (Figure 1d) [16].

### 2.2. Pulsatile Blood Flow Profiles

The pulsatile blood flow profile at the MV $U_{\mathrm{MV}}$ was based on a magnetic resonance imaging (MRI) velocity record [17] consisting of a biphasic wave; E and A wave peaks are associated with early rapid filling during ventricular relaxation and atrial contraction, respectively.

**Figure 2:**
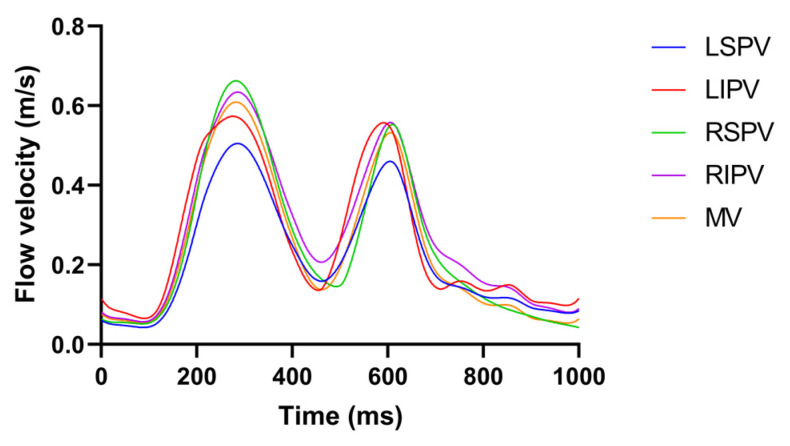
The pulsatile blood flow profiles at the four PVs and MV in the LA model.

The pulsatile blood flow profiles at the four PVs and MV in the LA model are shown in Figure 2, in which the characteristics of the pulsatile blood flow profiles at the four PVs (peak flow velocity, ratio of peak flow velocity, and time–velocity integral, etc.) and the mean LA flow velocity are within the range described in the literature [18,19].

### 2.3. Computational Study

This study used the AC/DC module, bio-heat transfer module, and computational fluid dynamics (CFD) module in COMSOL Multiphysics v5.6 (COMSOL, Stockholm, Sweden) to solve the electric field intensity distribution, temperature distribution generated by PFA in LA, and the cooling effect of the pulsatile blood flow in the cardiac chamber on the myocardium and the electrode during the application of PFA.

#### 2.3.1. Governing Equations

The electrical, thermal, and CFD equations involved in PFA in AF treatment are expressed as follows:

- Electrical Equations

The current density $J({A/m}^{2})$ and electric field intensity E(V/m) generated by PFA in LA are written as:

(1) $$J=\sigma_{e}\times E$$

(2) E=−∇V

where V represents the voltage and $\sigma_{e}(S/m)$ expresses the electrical conductivity, and the subindex e represents the electrical conductivity as AC or IC, e={a,i}, respectively.

No other electric source existed inside the LA, so its potential was regulated by the Laplace equation [20]:

(3) ∇⋅(σ∇V)=0

In LA, the power density $Q({W/m}^{3})$ at a given point is proportional to the electrical conductivity of the myocardium and the square of the magnitude of the electric field intensity vector:

(4) $$Q=\sigma_{m}\times {\left|E\right|}^{2}$$

Under the condition of AC, $\sigma_{a}$ can be considered as the second-order tensor of each node in the FEA, with [21]

(5) $$\sigma_{a}=\sigma_{t}I+\left(\sigma_{l}-\sigma_{t}\right)AA^{T}$$

where $\sigma_{t}$, $\sigma_{l}$, I, and A are the myocardial transverse electrical conductivity (perpendicular to the fiber orientation); longitudinal electrical conductivity (parallel to the fiber orientation); unit matrix; and direction cosine (α,β,γ) of the fiber orientation on each node acquired previously, respectively.

Therefore, on each node, $\sigma_{a}$ is expressed as:

(6) $$\sigma_{a}=\left|\begin{array}{ccc} \sigma_{t}+(\sigma_{l}-\sigma_{t})\alpha^{2} & (\sigma_{l}-\sigma_{t})\alpha \beta & (\sigma_{l}-\sigma_{t})\alpha \gamma \\ (\sigma_{l}-\sigma_{t})\alpha \beta & \sigma_{t}+(\sigma_{l}-\sigma_{t})\beta^{2} & (\sigma_{l}-\sigma_{t})\beta \gamma \\ (\sigma_{l}-\sigma_{t})\alpha \gamma & (\sigma_{l}-\sigma_{t})\beta \gamma & \sigma_{t}+(\sigma_{l}-\sigma_{t})\gamma^{2} \end{array}\right|$$

- Thermal Equations

The physical phenomena of the thermoelectric coupling problem are expressed by the Pennes biological heat transfer equation [22]:

(7) $$\rho c\frac{\partial T}{\partial t}=\nabla \cdot (k\nabla T)+Q+Q_{p}+Q_{m}-Q_{b}$$

where $\rho \left({\mathrm{kg}/m}^{3}\right)$ denotes the density; c(J/kg/K) represents the specific heat; k(W/m/K) is the thermal electrical conductivity; T is the temperature in K; $Q\left({W/m}^{3}\right)$ is the power absorption; $Q_{p}\left({W/m}^{3}\right)$ is the heat loss due to blood; and $Q_{m}\left({W/m}^{3}\right)$ is the metabolic heat generation. Since the contribution of the $Q_{p}$ and $Q_{m}$ is significantly smaller than those of other terms, it is neglected. $Q_{b}=\rho c\vec{u}\cdot \nabla T$ is the heat loss due to blood motion, where $\vec{u}(m/s)$ is the blood velocity field and is described by the incompressible Navier–Stokes equations consisting of the momentum and mass equations, as shown in the following:

- CFD Equations

(8) $$\rho \frac{\partial \vec{u}}{\partial t}+\rho \vec{u}\cdot \nabla \cdot \vec{u}=-\nabla P+\mu \nabla^{2}\vec{u}+F$$

(9) $$\nabla \cdot \vec{u}=0$$

where P(Pa) is the pressure, μ(kg/m/s) is the blood viscosity (μ = 2.1 × 10^−3^ kg/m/s), and $F({N/m}^{-3})$ is the body forces and is neglected in the LA model [23].

#### 2.3.2. Selection of Ablation Targets

**Figure 3:**
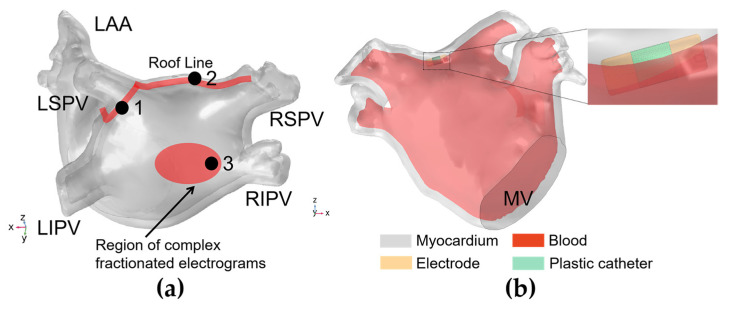
Ablation targets in the LA model and the ablation model. (**a**) Three representative ablation targets were selected based on the mainstream catheter ablation strategies for persistent AF in the LA model, corresponding to traditional pulmonary vein isolation (PVI) (ablation target 1), linear ablation (ablation target 2), and complex fractionated electrograms (CFAE) ablation (ablation target 3), respectively, where the LAA is the left atrial appendage, the LSPV/LIPV/RSPV/RIPV  is the left superior/left inferior/right superior/right inferior pulmonary vein, and the MV is  the mitral valve; (**b**) the ablation model where the catheter model is located at ablation target 2.

Figure 3a shows the three representative ablation targets selected based on the mainstream catheter ablation strategies for persistent AF in the LA model, corresponding to circumferential pulmonary vein isolation (PVI) (ablation target 1); linear ablation (ablation target 2); and complex fractionated electrograms (CFAE) ablation (ablation target 3), respectively [24,25]. Ablation target 1 is on the left superior pulmonary vein (LSPV) ostium, ablation target 2 is on the roofline in the middle of the LSPV and right superior pulmonary vein (RSPV), and ablation target 3 is on the LA posterior wall (PW) and is perpendicular to the right inferior pulmonary vein (RIPV) ostium. Among them, ablation targets 2 and 3 are selected based on the wide antral circumferential ablation (WACA) strategy, which is characterized by the catheter being placed ≥1.5 cm away from the PV ostium [26].

#### 2.3.3. Domain

The LA model was composed of myocardium and blood, and a catheter model was added in the LA endocardium consistent with the straight pressure catheter used in the actual PFA experiment. The catheter consists of two electrodes and is connected by an insulated plastic catheter in the middle; the diameter of the electrode and the plastic catheter is 7 F (1 F = 1/3 mm), and the length of the single electrode and the plastic catheter is 2 mm and 2.5 mm, respectively. To simulate the effect of slight electrode pressure on the endocardium, the catheter was inserted into the endocardium at a depth of 0.5 mm at all ablation targets and placed parallel to the epicardium to ensure consistent electrode–endocardium contact volume for all ablation target sites. The ablation model is illustrated in Figure 3b.

#### 2.3.4. Boundary Conditions

- Electrical Boundary Conditions

**Figure 4:**
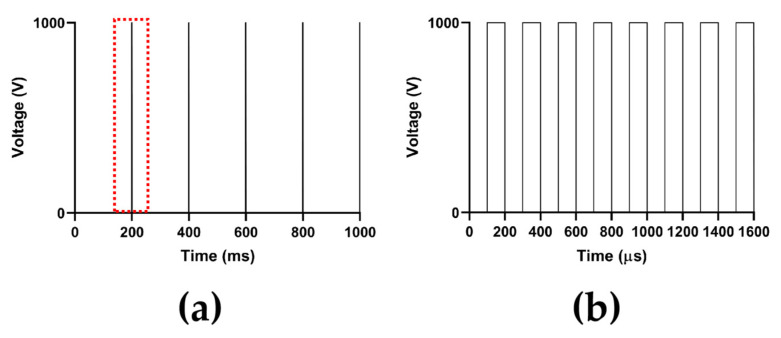
The waveform of P(t) in the computation of the temperature distribution, where (**a**) is the complete waveform of P(t) with a total duration of 1 s, and (**b**) is the detail of the complete waveform of a single pulse train in the red box of (**a**).

The biphasic pulsed voltage $V_{a}=P(t)$ was applied on the left electrode, and the zero voltage was applied on the right electrode; the boundary of the plastic catheter and the epicardium represented the zero electric flux conditions and is expressed as:

(10) n×σ∇V=0

where n denotes the unit vector. The parameters of the P(t) are as follows:

1. In the computation of the electric field intensity distribution, the amplitude of the P(t) is selected as 1000 V, 1500 V, and 2000 V, respectively, to cover the amplitude output range of PFA generators used in AF treatment and the animal experiment [27,28].
2. In the computation of the temperature distribution, the amplitude of the P(t) is 1000 V, and the total PFA duration is 1 s, containing five pulse trains and five pulse train intervals; each pulse train contains eight pulses and eight pulse intervals, each pulse width and pulse interval are both 100 μs, and the pulse train interval is 198.4 ms, to consist with the parameters of PFA generators used in the animal experiment [28]. The waveform of P(t) in the computation of the temperature distribution is shown in Figure 4.

- Thermal Boundary Conditions

Thermal boundary conditions comprised an initial temperature boundary condition and one type of thermal boundary condition:

1. Initial temperature boundary condition: At *t* = 0, the initial temperature of the myocardium *T*_0_ and the blood *T*_b_ was set to 37 °C.
2. The second type of thermal boundary condition (constant heat flux boundary condition): The boundary of the plastic catheter and the epicardium represented the zero heat flux conditions and is expressed as:

(11) n×k∇T=0

- CFD Boundary Conditions

A no slip condition was applied on the myocardium–blood and electrode–blood interfaces. For fluid dynamics, the blood flow in the LA was simulated by setting the four PVs as inflows with a condition of 10 mmHg [29], and the MV as an outflow with a pulsatile blood flow profile $U_{\mathrm{MV}}$ (see “Section 2.2 Pulsatile Blood Flow Profiles”).

The fluid type was determined by calculating the Reynolds number in the PV as:

(12) Re=ρu→Dμ

where $\rho ({\mathrm{kg}/m}^{3})$ is the blood density, D(m) is the diameter of the PV. As the Reynolds number for the peak blood flow velocities in the four PVs and MV are all less than 2000, the fluid type was set as laminar flow.

**Figure 5:**
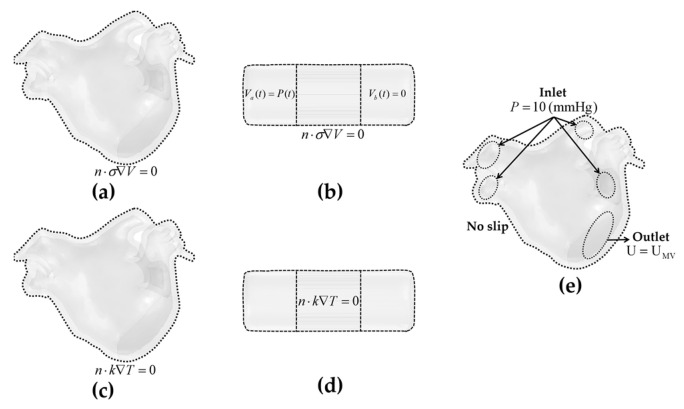
The electrical boundary conditions, thermal boundary conditions, and CFD boundary conditions of the ablation model. Electrical boundary conditions of (**a**) the LA model and (**b**) the catheter model; thermal boundary conditions of (**c**) the LA model and (**d**) the catheter model; and CFD boundary conditions of (**e**) the LA model.

Figure 5 illustrates the boundary conditions of the ablation model.

#### 2.3.5. Material Properties

The electrical and thermal properties of the ablation model are listed in Table 1, and detailed AC and the corresponding IC of the myocardium are listed in Table 2.

Before applying PFA, the cell membrane is intact and acts as an electrical insulator; the electrical conductivity of the tissue corresponds to that of low frequency, while after the PFA-induced creation of the pores, the pores in the cell membrane allow the passage of electric current through the cytoplasm and the extracellular space. Hence, the cell becomes more permeable to electrical currents, and the electrical conductivity of the tissue is similar to that of high frequency. It is usual to use a sigmoid function that relates the electrical conductivity of the tissue (for example, the myocardium) to the electric field magnitude during PFA and is expressed as [30]:

(13) $$\sigma (E)=\sigma_{0}+\frac{\sigma_{1}-\sigma_{0}}{1+10e^{-\frac{\left(|E|-58,000\right)}{3000}}}$$

where $\sigma_{0}$ and $\sigma_{1}$ are the pre- and post-electroporation myocardial electrical conductivities, respectively. Since no specific data are currently available for the myocardium, this study chose the electrical conductivity values at 10 Hz and 500 kHz to simulate cardiac cells pre- and post-electroporation, i.e., $\sigma_{0}=0.0537\;S/m$ and $\sigma_{1}=0.281\;S/m$, respectively [30,31].

In this study, $\sigma_{0}$ is considered as myocardial IC ($\sigma_{0}=\sigma_{i}$), four groups of myocardial AC corresponding to IC are obtained according to the various anisotropy ratios R, and the IC is obtained by averaging the AC in the transverse and longitudinal directions [21], i.e.,

(14) $$\{\begin{array}{c} \sigma_{l}/\sigma_{t}=R\\ \left(\sigma_{l}+\sigma_{t}\right)/2=\sigma_{i} \end{array}$$

to compare the effect of AC with a different size and anisotropy ratio on the ablation characteristics generated by PFA in LA. According to the type of myocardium, the anisotropy ratio of myocardial electrical conductivity in groups 1 and 2 are derived from the literature on normal myocardium, and groups 3 and 4 are derived from the literature on myocardium with AF. Compared with the normal myocardial electrical conductivity, in the myocardium with AF electrical conductivity, the formation of fibrosis in the myocardium leads to the increase in the anisotropy ratio of myocardial conduction velocity, which further leads to the increase in the anisotropy ratio of myocardial electrical conductivity [32]. Therefore, as shown in Table 2, the anisotropy ratios of myocardial electrical conductivity in groups 3 and 4 are larger than those in groups 1 and 2.

### 2.4. Statistical Analysis

Data are processed using SPSS v20.0 (IBM, Armonk, NY, USA) and are expressed as mean ± standard deviation. The size of the ablation isosurface is in mm, and the difference in size between AC and IC is the absolute value. The size of the surface ablation area is in square mm, and the percentage difference in the size of the surface ablation area between AC and IC is calculated as ${(S}_{\mathrm{AC}}-{\;S}_{\mathrm{IC}}{)/S}_{\mathrm{IC}}\times 100\%$. The size of the ablation volume is in cubic mm, and the percentage difference in the size of the ablation volume between AC and IC is calculated as ${(V}_{\mathrm{AC}}-V_{\mathrm{IC}}{)/V}_{\mathrm{IC}}\times 100\%$.

This study considered that the differences in the size of the ablation isosurface are insignificant for values less than 1 mm, since this value is approximate to the deviation (±0.5 mm) observed in experimental studies on RFA [35]. This study also considered that the percentage differences in the size of the surface ablation area and ablation volume between AC and IC are insignificant for values less than 5%, since this value is almost negligible in clinical terms. Likewise, the differences in the maximum temperature reached in the myocardium between AC and IC are considered insignificant for values less than 4 °C, since this value is approximate to the observed deviation (±2 °C) [35].

## 3. Results

### 3.1. Estimated Fiber Orientation

**Figure 6:**
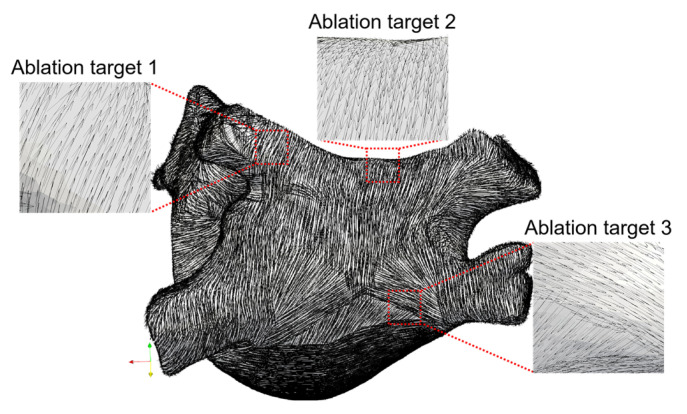
The fiber orientation incorporated into the LA model and the details of the fiber orientation at three ablation targets.

According to Equation (6), the AC is essentially reflected by the distribution of fiber orientation throughout the entire LA; therefore, the correctness of the fiber orientation incorporated into the LA model is crucial for the subsequent investigation. In this study, the generated fiber orientation conforms to the realistic fiber orientation based on the descriptions in the literature and histological observations [36,37,38]. Figure 6 shows the fiber orientation incorporated into the LA model and the details of the fiber orientation at three ablation targets.

As shown in Figure 6, circumferential fibers are observed surrounding the PVs (ablation target 1), and the openings of the PVs are crossed by fibers running in various directions [36]. The septopulmonary bundle oblique passes the LA roof (ablation target 2) and fans out to pass the insertions of the PVs. On the PW, the septopulmonary bundle becomes two diverging branches, and the septoatrial bundle located in the endocardium is revealed; both rejoin in the circumferential myofibers from the lateral wall obliquely (ablation target 3) [37,38].

### 3.2. Surface Ablation Area

The previous section shows significant differences in shape and fiber orientation at each ablation target; these two factors will lead to the difference in the electric field intensity distribution generated by PFA under the condition of the same electrical conductivity at each ablation target. Presently, a specific electric field intensity threshold is usually used to estimate whether PFA causes effective myocardial ablation [1,3,4,39]; this study used the IRE threshold for cardiac cells, which is defined as the electric field intensity exceeding 1000 V/cm [40].

**Figure 7:**
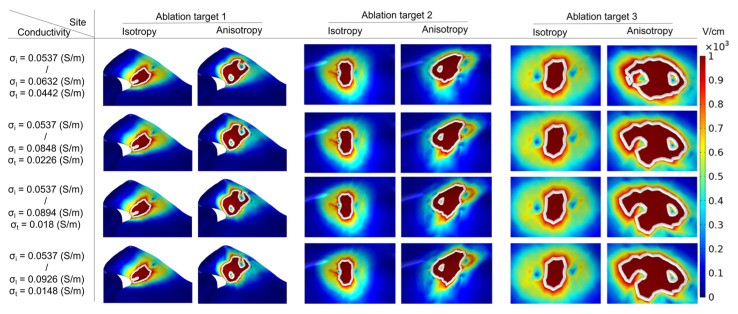
Under the condition of the PFA, the amplitude is 1000 V, and the electric field intensity distribution with ablation isoline is generated by different AC and IC at three ablation targets, where solid white lines represent the ablation isoline and are defined as the electric field intensity equal to 1000 V/cm.

To determine the surface ablation area, this study first created the surface and isoline in the result of the electric field intensity distribution in COMSOL and set the value of isoline to 1000 V/cm, then selected the different plane views for each ablation target (ablation target 1: X-Z plane, 2: X-Y plane, 3: X-Z plane). Finally, this study enlarged and adjusted the view of the electric field intensity distribution at each ablation target under the condition of different groups of electrical conductivity, and the magnification of the view is the same in all cases. Figure 7 shows that under the condition of the PFA, the amplitude is 1000 V, and the electric field intensity distribution with ablation isoline (1000 V/cm) is generated by different ACs and ICs at three ablation targets.

**Figure 8:**
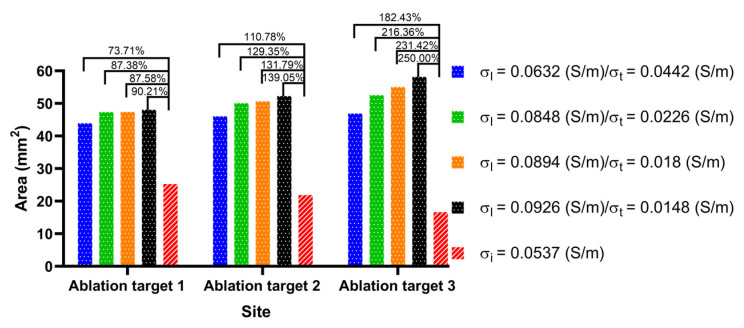
The statistical results of the size of the surface ablation area under the condition of the PFA amplitude is 1000 V at three ablation targets, where the numbers between the top of the bars represent the absolute percentage difference in the size of the surface ablation area between AC and IC in the four electrical conductivity groups, respectively.

To determine the size of the surface ablation area, this study used the surface integral function built into COMSOL to integrate the area with electric field intensity greater than 1000 V/cm in the surface of the LA model. Figure 8 shows that the statistical results of the size of the surface ablation area under the condition of the PFA amplitude is 1000 V at each ablation target, respectively.

The electrical conductivity groups in Figure 7 are arranged in ascending order of anisotropy ratio from top to bottom. As shown in Figure 7, at the same ablation target, the differences in the surface ablation area generated by AC compared with IC are distinguishable. Under the condition of a different AC, with the increase in the anisotropy ratio, the surface ablation area tends to extend towards the fiber orientation at the ablation target. According to Figure 8, under the condition of the same ablation target, the sizes of the surface ablation area generated under the condition of AC are all larger than IC, and with the increase in the anisotropy ratio, the difference in the size of the surface ablation area generated by AC compared with IC increases at each ablation target. The percentage differences in the size of the surface ablation area between AC and IC are significant, since the values are greater than 73.71% in all cases.

Specifically, at ablation target 1 the catheter is placed parallel to the LSPV ostium. Under the condition of IC, the surface ablation area extends along the catheter to both sides of the LSPV ostium. Under the condition of AC, with the increase in the anisotropy ratio, the surface ablation area near the positive electrode gradually extends obliquely towards the top of LSPV along the circumferential fibers.

At ablation target 2, the catheter is placed parallel to the LA roof. Under the condition of IC, the surface ablation area is perpendicular to the LA roof. Under the condition of AC, with the increase in the anisotropy ratio, the surface ablation region located at the plastic catheter gradually extends obliquely towards the PW along the direction of the septopulmonary bundle, which is located on the LA roof.

At ablation target 3, the catheter is placed on the PW and is perpendicular to the RIPV ostium. Under the condition of IC, the surface ablation region is primarily perpendicular to the plastic catheter. Under the condition of AC, the surface ablation area located in the upper part of the catheter gradually extends to the left of the PW along the direction of the septopulmonary bundle branch, and the lower part of the catheter gradually extends to the lower left of the PW along the direction of the septoatrial bundle.

### 3.3. Ablation Isosurface and Ablation Volume

The previous section shows that AC significantly impacts the surface ablation area more than IC at each ablation target generated by PFA in LA. This study further investigates the ablation isosurface and ablation volume generated by AC compared with IC at each ablation target; the definition of the ablation isosurface is the same as before (1000 V/cm).

#### 3.3.1. Ablation Isosurface

**Figure 9:**
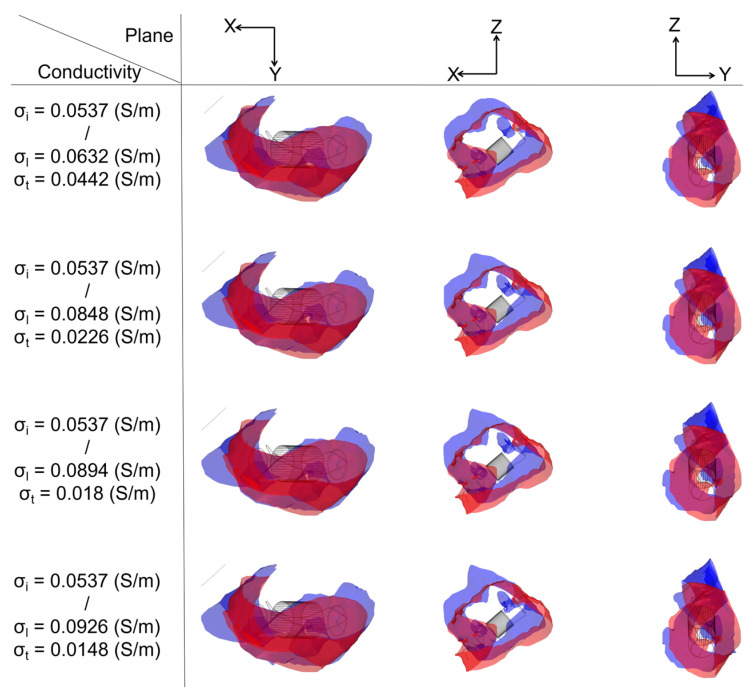
Under the condition of the PFA, the amplitude is 1000 V; at ablation target 1 the ablation isosurface distribution is generated by different ACs and ICs, where the ablation isosurface is defined as the electric field intensity equal to 1000 V/cm. The red area and blue area represent the ablation isosurface generated by IC and AC, respectively.

To determine the ablation isosurface, this study first created the isosurface in the result of the electric field intensity distribution in COMSOL and set the value of isosurface to 1000 V/cm, then adjusted the view and obtained isosurfaces of IC and each group of AC in three different plane views (X-Y plane, Y-Z plane, and X-Z plane) under the condition of a different ablation target; finally, the study exported the isosurfaces and superimposed the isosurfaces of IC on the isosurfaces of each group of AC in three different plane views based on the position of the catheter. Figures 9, 11 and 13 show that under the condition of the PFA the amplitude is 1000 V, and the ablation isosurface (1000 V/cm) distribution is generated by different ACs and ICs at each ablation target, respectively.

**Figure 10:**
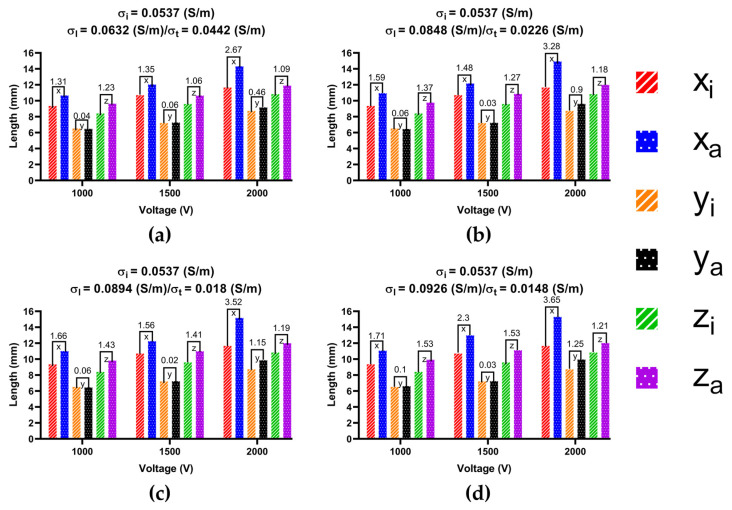
The statistical results of the size of the ablation isosurface under the condition of different PFA amplitudes at ablation target 1, where the subscript i and a of each legend represent the three geometric ablation sizes (*X*-axis, *Y*-axis, *Z*-axis) generated by IC and AC, respectively, and the numbers in mm between the top of the bars represent the absolute difference in the size of the ablation isosurface between AC and IC in three ablation geometric sizes, respectively. The statistical results of the size of the ablation isosurface in myocardial electrical conductivity (**a**) group 1; (**b**) group 2; (**c**) group 3; and (**d**) group 4.

To determine the size of the ablation isosurface, this study first exported the coordinate data of the isosurfaces of IC and each group of AC under the condition of different PFA amplitudes and ablation targets, then sorted the coordinates of the *X*-axis, *Y*-axis, and *Z*-axis of each isosurface, respectively, and finally obtained the difference between the maximum and minimum of the three axes as the three sizes of each isosurface. Figures 10, 12 and 14 show the statistical results of the size of the ablation isosurface under the condition of different PFA amplitudes at each ablation target, respectively.

**Figure 11:**
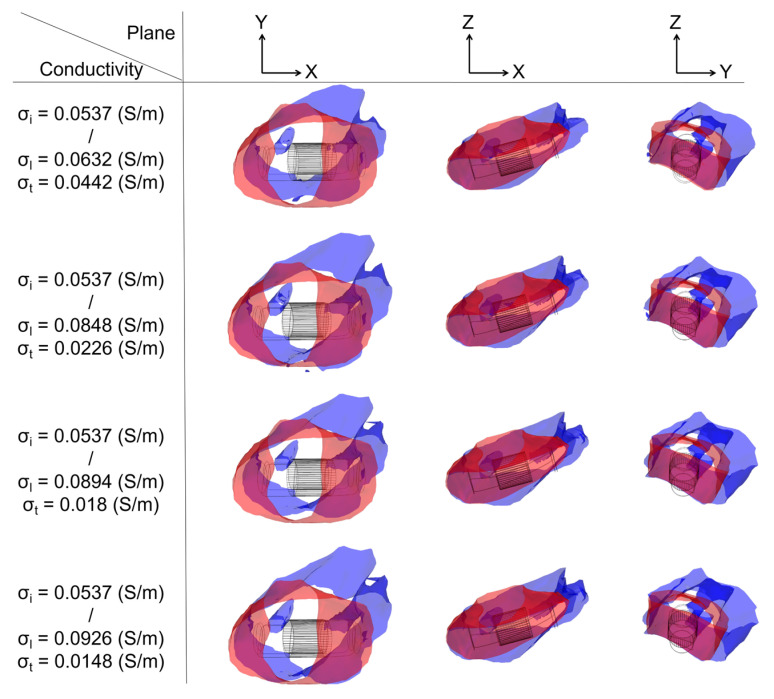
Under the condition of the PFA, the amplitude is 1000 V; at ablation target 2, the ablation isosurface distribution is generated by different ACs and ICs.

**Figure 12:**
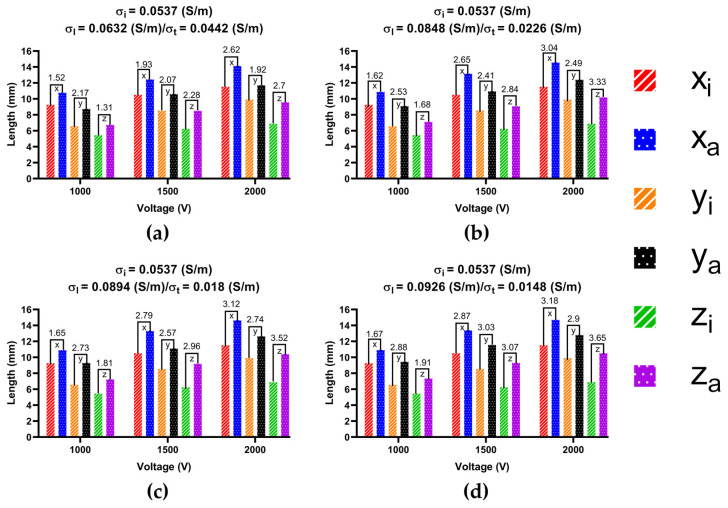
The statistical results of the size of the ablation isosurface under the condition of different PFA amplitudes at ablation target 2. The statistical results of the size of the ablation isosurface in myocardial electrical conductivity (**a**) group 1; (**b**) group 2; (**c**) group 3; and (**d**) group 4.

**Figure 13:**
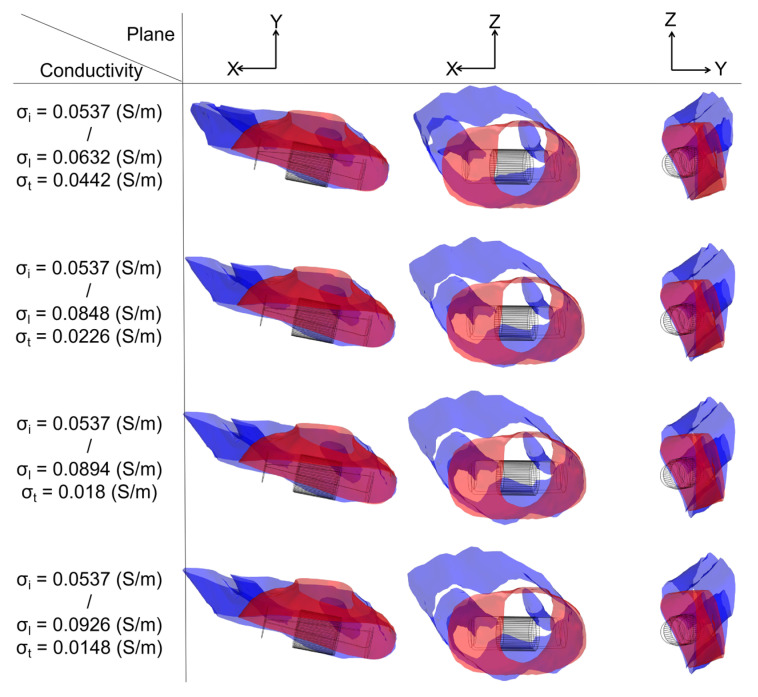
Under the condition of the PFA, the amplitude is 1000 V; at ablation target 3, the ablation isosurface distribution is generated by different ACs and ICs.

**Figure 14:**
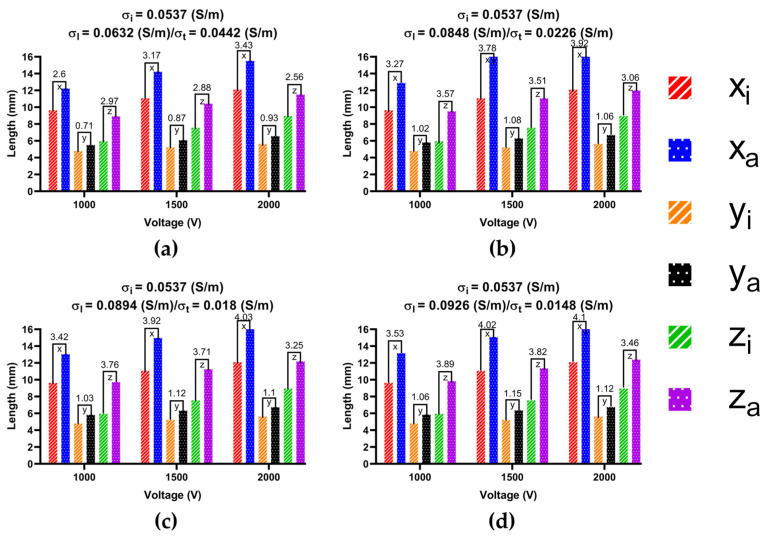
The statistical results of the size of the ablation isosurface under the condition of different PFA amplitudes at ablation target 3. The statistical results of the size of the ablation isosurface in myocardial electrical conductivity (**a**) group 1; (**b**) group 2; (**c**) group 3; and (**d**) group 4.

As shown in Figures 9, 11 and 13, with the increase in the anisotropy ratio, the ablation isosurface generated by AC tends to extend slightly towards the fiber orientation at the ablation target; as a result, the difference in the ablation isosurface generated by AC compared with IC becomes larger. As shown in Figures 10, 12 and 14, under the condition of the same electrical conductivity, the PFA amplitude is highly linearly positively correlated with the generated three sizes (*X*-axis, *Y*-axis, and *Z*-axis) (correlation coefficient > 99%) at each ablation target, and under the condition of the same electrical conductivity and PFA amplitude, the three sizes of the ablation isosurfaces generated under the condition of AC are almost all larger than IC.

Specifically, as shown in Figure 9, with the increase in the anisotropy ratio, the ablation isosurface extends obliquely towards the top of LSPV along the circumferential fibers. According to Figure 10, except for under the condition of the PFA, the amplitude is 2000 V. The AC and the corresponding IC from the third and fourth electrical conductivity group, as well as the size differences in the *Y*-axis between AC and IC are all greater than 1 mm; in other conditions, the size differences in the *Y*-axis between the two are less than 1 mm. The size differences in the *X*-axis and *Z*-axis between AC and IC are greater than 1 mm in all cases. Overall, the mean difference in the size of the ablation isosurface in the four electrical conductivity groups is 2.17 ± 0.88 mm (*X*-axis), 0.35 ± 0.48 mm (*Y*-axis), and 1.29 ± 0.16 mm (*Z*-axis), respectively, and the maximum difference is 3.65 mm (*X*-axis), 1.25 mm (*Y*-axis), and 1.53 mm (*Z*-axis), respectively.

As shown in Figure 11, at ablation target 2, with the increase in the anisotropy ratio, the ablation isosurface extends obliquely towards the PW along the direction of the septopulmonary bundle located on the LA roof. According to Figure 12, the size differences in the *X*-axis, *Y*-axis, and *Z*-axis between AC and IC are greater than 1 mm in all cases. Overall, the mean difference in the size of the ablation isosurface in the four electrical conductivity groups is 2.39 ± 0.65 mm (*X*-axis), 2.54 ± 0.35 mm (*Y*-axis), and 2.59 ± 0.78 mm (*Z*-axis), respectively, and the maximum difference is 3.18 mm (*X*-axis), 3.03 mm (*Y*-axis), and 3.65 mm (*Z*-axis), respectively.

As shown in Figure 13, at ablation target 3, with the increase in the anisotropy ratio, the ablation isosurface extends obliquely towards the upper left of the PW along the direction of the septopulmonary bundle. According to Figure 14, except for under the condition of the first electrical conductivity, the size differences in the *Y*-axis between AC and IC are less than 1 mm; in other conditions, the size differences in the *Y*-axis between the two are greater than 1 mm. The size differences in the *X*-axis and *Z*-axis between AC and IC are greater than 2 mm in all cases. Overall, the mean difference in the size of the ablation isosurface in the four electrical conductivity groups is 3.6 ± 0.45 mm (*X*-axis), 1.02 ± 0.13 mm (*Y*-axis), and 3.37 ± 0.42 mm (*Z*-axis), respectively, and the maximum difference is 4.03 mm (*X*-axis), 1.15 mm (*Y*-axis), and 3.89 mm (*Z*-axis), respectively.

#### 3.3.2. Ablation Volume

**Figure 15:**
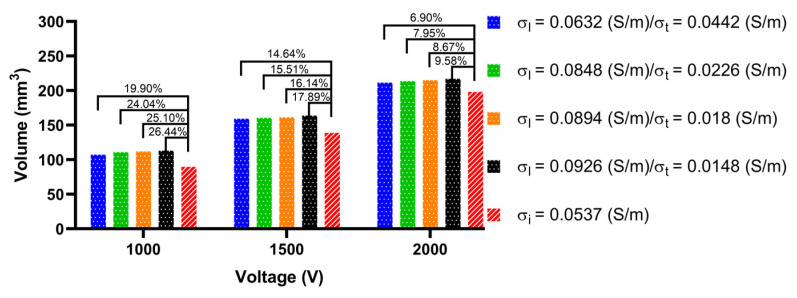
The statistical results of the size of the ablation volume generated by different ACs and ICs under the condition of different PFA amplitudes at ablation target 1, where the numbers between the top of the bars represent the absolute percentage difference in the size of the ablation volume between AC and IC in the four electrical conductivity groups, respectively.

**Figure 16:**
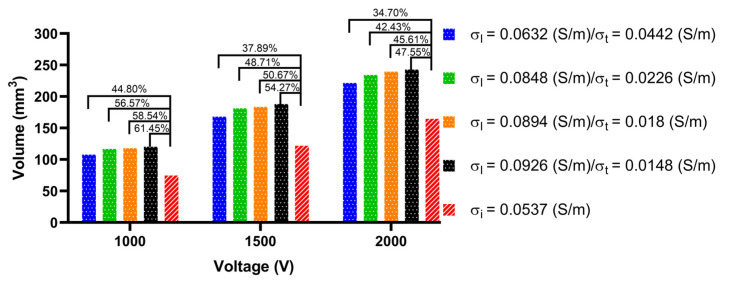
The statistical results of the size of the ablation volume generated by different ACs and ICs under the condition of different PFA amplitudes at ablation target 2.

**Figure 17:**
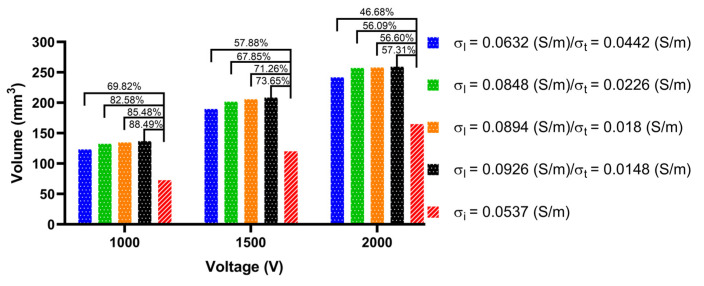
The statistical results of the size of the ablation volume generated by different ACs and ICs under the condition of different PFA amplitudes at ablation target 3.

To obtain the size of the ablation volume, this study used the volume integral function built into COMSOL to integrate the area with an electric field intensity greater than 1000 V/cm in the myocardial domain of the LA model. Figure 15, Figure 16 and Figure 17 show the statistical results of the size of the ablation volume generated by different AC and IC under the condition of different PFA amplitudes at each ablation target, respectively.

As shown in Figure 15, Figure 16 and Figure 17, under the condition of the same electrical conductivity, the PFA amplitude is highly linearly positively correlated with the generated ablation volume (correlation coefficient > 99%) at each ablation target. Under the condition of the same PFA amplitude, the sizes of the ablation volume generated under the condition of AC are all larger than IC, and with the increase in the anisotropy ratio, the difference in the size of the ablation volume generated by AC compared with IC increases at each ablation target. The percentage differences in the size of the ablation volume between AC and IC are significant, since the values are greater than 6.9% in all cases.

In terms of the type of myocardial electrical conductivity, under the condition of the same PFA amplitude, at each ablation target the ablation volumes generated by the AC of the normal myocardial electrical conductivity groups are all smaller than that of the myocardium with AF electrical conductivity groups.

In terms of the size of the ablation volume, under the condition of different PFA amplitudes, the mean percentage difference of the ablation volume generated by AC compared with IC amplitude at each ablation target is 152.19 ± 45.45 mm^3^ (ablation target 1), 148.64 ± 52.86 mm^3^ (ablation target 2), and 157.54 ± 59.99 mm^3^ (ablation target 3).

In terms of the difference in ablation volume, with the increase in the anisotropy ratio, under the condition of different PFA amplitudes, the mean percentage difference of the ablation volume generated by AC compared with the IC amplitude at each ablation target is 16 ± 7% (ablation target 1), 49 ± 8% (ablation target 2) and 68 ± 13% (ablation target 3), respectively, and the maximum percentage difference is 26.44% (ablation target 1), 61.45% (ablation target 2) and 88.49% (ablation target 3), respectively.

### 3.4. Temperature Increase

**Figure 18:**
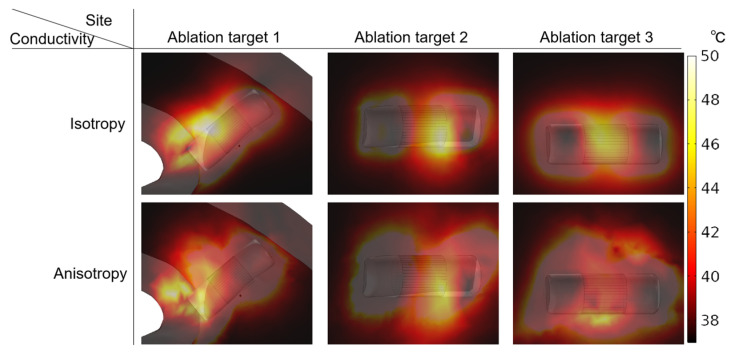
Under the condition of the same PFA parameters (see “Boundary Conditions”) and AC and the corresponding IC from the fourth electrical conductivity group with the highest anisotropy ratio, the final temperature distribution is generated in the myocardium at three ablation targets.

**Figure 19:**
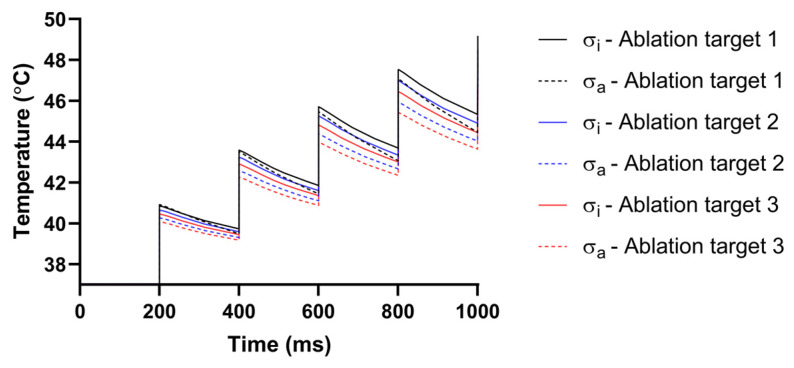
Under the condition of the same PFA parameters (see “Boundary Conditions”) and AC and the corresponding IC from the fourth electrical conductivity group with the highest anisotropy ratio, the curve of maximum temperature is generated in the myocardium at three ablation targets.

In a review of relevant studies, the temperature distribution under the condition of AC compared with IC generated by PFA in LA has not been investigated. To investigate the influence of AC and IC on temperature increase, this study compares the temperature under the condition of the same PFA parameters (see “Boundary Conditions”) and AC and the corresponding IC from the fourth electrical conductivity group with the highest anisotropy ratio, the final temperature distribution generated in the myocardium at three ablation targets (Figure 18), and the curve of maximum temperature generated in myocardium at three ablation targets (Figure 19), respectively.

As shown in Figure 18, in all cases, the highest temperature area in the myocardium is located where the positive electrode is in contact with the myocardium. Similar to the electric field distribution, the temperature distribution generated by IC is more uniform than that of AC at each ablation target, and the temperature distribution under the condition of AC tends to extend towards the fiber orientation at the ablation target. Although the temperature increases generated by AC are smaller than IC at each ablation target, the differences in the temperature increase at each ablation target between AC and IC are insignificant, since the values are all less than 2.46 °C.

As shown in Figure 19, although the maximum temperature in the myocardium increases rapidly for a short time during the application of each pulse train, due to the cooling effect of the pulsatile blood flow in the cardiac chamber on the myocardium and the electrode, the maximum temperature decreases slowly during each subsequent pulse train interval. Finally, although the temperature increase at ablation target 1 is the most obvious under the condition of IC, the highest temperature in the myocardium is 49.18 °C.

## 4. Discussion

### 4.1. Importance of Fiber Orientation

In the computational studies of PFA in AF treatment, to shorten the model construction process and reduce the computational complexity, the LA model is usually defined as a regular shape model such as a sphere [39] or a cylinder [41], and the fiber orientation is ignored; or the LA model is based on 3D medical imaging reconstruction [3,4] and the fiber orientation is ignored. Although Xie et al. [1] constructed a myocardial model composed of multi-layer circular slabs and simulated fiber orientation by assigning an AC tensor to each layer, such regular fiber orientation still cannot reflect the influence of realistic fiber orientation on ablation characteristics in PFA in AF treatment consistently.

Since the atrial wall is significantly thinner than the ventricular wall, and atrial fiber architecture is highly complex [42], the main reason for neglecting fiber orientation in the construction of the LA model in relevant studies is that it has so far been complex and time-consuming. Although it could be possible to extract the fiber orientation of excised human atrium using diffusion tensor magnetic resonance imaging or micro-computed tomography, to obtain fiber orientation successfully, the former requires complex tissue preparation and image post-processing [43], while the latter requires higher image resolution and quality [42]. Therefore, extracting fiber orientation from the medical image of the atrium still has a high threshold and is not easy to obtain.

To investigate the effect of AC induced by fiber orientation on the ablation characteristics of PFA in AF treatment, this study used a robust semi-automatic method to incorporate fiber orientation into the patient-specific LA model reconstructed by CTA images; compared with the above method, this method significantly minimizes the challenge of incorporating fiber orientation into the LA. The defect of defining LA as a regular shape model and/or ignoring fiber orientation is that it cannot reflect the effect of realistic LA shape and fiber orientation on the electric field intensity and temperature distribution generated by PFA in LA. From the results of this study, it can be concluded that both influence the electric field intensity and temperature distribution, which tends to extend towards the fiber orientation at the ablation target. Therefore, to reflect the actual ablation results as consistently as possible, constructing the anatomy-based LA model incorporating fiber orientation may be the correct trend for further studies in the computational study of PFA in AF treatment, and an essential step to introducing the reliable patient-specific LA model into clinical practice.

### 4.2. Effect of Different Ablation Targets

Most triggers for paroxysmal AF come from the PVs, so ablation involves creating circumferential lesions around the PVs to isolate them from the rest of the LA electrically. Catheter ablation for persistent AF is more challenging and has less favorable outcomes. To improve these outcomes, ablation targeting the substrate that maintains AF (i.e., substrate modification) is often added to PVI; the two most common techniques for substrate modification are the creation of linear lesions in the LA and focal ablation to eliminate atrial signals that show CFAE [24]. Guidelines also suggest that “operators should consider more extensive ablation based on linear lesions or CFAE” for the ablation of persistent AF [25].

In terms of the distance from the ablation target to the PV ostium, ablation target 1 belongs to the circumferential PVI strategy, and the distance from the catheter to the PV ostium is in the range of 0.5–1 cm, which requires complete circumferential ablation of the LA around the upper and lower PVs on both sides [44]. Ablation targets 2 and 3 are selected based on the WACA strategy, which is characterized by the catheter being placed ≥1.5 cm away from the PV ostium [26]. Proietti et al. [26] considered that PVI performed with a WACA strategy is more effective than ostial PVI in achieving freedom from total atrial tachyarrhythmia recurrence at long-term follow-up; it also has the potential advantage of ablating non-PV foci (e.g., CFAE) that are localized in the LA PW.

### 4.3. Effect of Different Electrical Conductivity

According to the type of myocardium, the myocardium electrical conductivity selected in this study can be divided into the normal myocardial electrical conductivity and myocardium with AF electrical conductivity. Compared with the normal myocardial electrical conductivity, in the myocardium with AF electrical conductivity, the formation of fibrosis in the myocardium leads to the increase in the anisotropy ratio of myocardial conduction velocity, which further leads to the increase in the anisotropy ratio of myocardial electrical conductivity [32]. As shown in the results of this study, the ablation volumes generated by the normal myocardial electrical conductivity are smaller than that of the myocardium with AF electrical conductivity at each ablation target; that is, the anisotropy ratio of the myocardial electrical conductivity is positively correlated with the generated ablation volume. Therefore, to generate contiguous and transmural ablation in PFA in AF treatment, PFA energy should be adjusted reasonably according to the degree of myocardial fibrosis.

In terms of ablation characteristics, IC ignores the influence of fiber orientation, resulting in the surface ablation area and ablation isosurface generated by IC being more uniform than that of AC at each ablation target. Under the condition of AC, the above two ablation characteristics are affected by the fiber orientation and tend to extend towards the fiber orientation at the ablation target; at the same time, the myocardial fiber orientation is usually highly concentrated in a specific direction at the ablation target, resulting in the percentage differences in the size of the surface ablation area and ablation volume between AC and IC being greater than 5% in all cases, and the maximum differences in the size of the ablation isosurface between AC and IC are all greater than 1 mm at each ablation target. Hence, this study suggests that in future research on the computational study of PFA in AF treatment, to obtain more accurate computational results, it is necessary to adopt AC rather than IC to investigate the size of the surface ablation area, the size of the ablation isosurface, or the size of the ablation volume generated by PFA in LA.

At the same time, due to using the function of electrical conductivity, which changes with electric field magnitude, in each group of AC with a different anisotropy ratio, the difference between the myocardial transverse and longitudinal electrical conductivity after electroporation is reduced, and both of them are close to the post-electroporation electric conductivity $\sigma_{1}$, since the myocardial electrical conductivity increases significantly after electroporation. Hence, with the increase in the anisotropy ratio, the difference in the size of the ablation isosurface generated by each group of AC are all insignificant, since the values between the size of the three axes of the ablation isosurfaces of AC generated under the same PFA amplitude and different electrical conductivity groups are all less than 1 mm, respectively.

In terms of temperature increase, under the conditions of specific PEF parameters applied in this study (see “Boundary Conditions”), the difference in the temperature increase between AC and IC in LA are insignificant in all cases. Hence, this study suggests that in the future research on the computational study of PFA in AF treatment, if only investigating the temperature increase generated by PFA in LA, adopting IC instead of AC for simplifying the model construction process is reasonable.

### 4.4. The Differences of Male/Female in Model Construction

The anatomy-based LA model constructed by CTA images in this study is from a female patient. In a review of relevant studies, no studies have shown that the LA fiber orientation of the males is significantly different from that of the females. Although the mean LA volume is slightly larger in males than in females in most humans, the difference is not statistically significant [45].

Therefore, this study preliminarily considers that if we happen to use an LA model of a male patient with an LA volume slightly larger than the current LA model, then the shape and the size of the surface ablation area and ablation isosurface will be changed somewhat compared with the result in the current study, because in LAs with a larger volume, the curvature of the LA wall where the catheter is attached is smaller.

### 4.5. Compared with the Experimental Results

The electrode direction relative to the fiber orientation of tissue has proved to have a significant effect on the ablation effect; in general, when the electrode is parallel to the fiber orientation of tissue, the generated ablation area is larger than that which is perpendicular to the fiber orientation of tissue [46,47]. The above studies support the reliability of the computational results in this study to a certain extent.

### 4.6. Limitations

This study also had some limitations. The most prominent limitation is that we did not use the experimental results to support the reliability of the computational results in this study. In fact, the ablation of living rabbit myocardium using the PFA generator and the straight pressure catheter was originally part of this study plan; however, due to the impact of COVID-19, we have so far been unable to carry out this experiment. 

The second limitation is that the results and conclusions in this study are based on specific myocardial electrical conductivity, pulse parameters, and electric field intensity damage threshold. However, to date, the myocardial electrical conductivities are not well characterized for low frequencies (as is the case for PFA) since they are based on estimations of those obtained experimentally above 1 MHz [48]. The PFA parameters used in the AF treatment and the animal experiment vary within a certain range, and the cardiac cell electric field damage threshold varies with PFA parameters within a certain range [49].

The third limitation is that fiber orientation changes depending on the atrial depth in different atrial areas; however, due to the limitation of the current semi-automatic algorithms for generating fiber orientation into the patient-specific atrial model, we cannot investigate the influence of this factor on the ablation characteristics of PFA in AF treatment.

The fourth limitation is that the ablation model constructed in this study is macro and does not include the myocardial cell models, so it is impossible to investigate the influence of other PFA parameters other than the PFA amplitude on ablation characteristics.

## 5. Conclusions

This study constructed an anatomy-based LA model incorporating fiber orientation and selected various electrical conductivity and ablation targets to investigate the effect of AC compared with IC on the ablation characteristics of PFA in AF treatment. According to quantifications, the results show that the difference in the size of the surface ablation area, the size of the ablation isosurface, and the size of the ablation volume between AC and IC are significant; however, the difference in the temperature increase between the two are insignificant. Hence, this study suggests that in further studies in the computational study of PFA in AF treatment using the same or similar conditions as those used in this study (myocardial electrical conductivity, pulse parameters, and electric field intensity damage threshold), to obtain more accurate computational results, it is necessary to adopt AC rather than IC to investigate the size of the surface ablation area, the size of the ablation isosurface, or the size of the ablation volume generated by PFA in LA. Moreover, if only investigating the temperature increase generated by PFA in LA, adopting IC instead of AC for simplifying the model construction process is reasonable.

## Figures and Tables

**Table 1:** The electrical and thermal properties of the ablation model [21,33].

Element/Material	$\rho ({\mathrm{kg}/m}^{3})$	c(J/kg/K)	k(W/m/K)	σ(S/m)
Electrode	21,500	132	71	4.6 × 10^6^
Plastic Catheter	70	1045	0.026	1 × 10^−5^
Blood	1000	4180	0.54	0.99
Myocardium	1200	3200	0.53	Table 2

**Table 2:** Four groups of myocardial AC and the corresponding IC.

Group/Electrical Conductivity	AC			IC	Reference
	$\sigma_{l}(S/m)$ *	$\sigma_{t}(S/m)$ **	R ***	$\sigma_{i}(S/m)$ ****	
1	0.0632	0.0442	1.429	0.0537	[21]
2	0.0848	0.0226	3.75	0.0537	[6]
3	0.0894	0.018	4.98	0.0537	[7]
4	0.0926	0.0148	6.25	0.0537	[34]

* Longitudinal myocardial electrical conductivity; ** Transverse myocardial electrical conductivity; *** The anisotropy ratio of myocardial electrical conductivity; **** Isotropic myocardial electrical conductivity.

## Data Availability

The datasets used and/or analyzed during the current study are available from the corresponding author on reasonable request.
